# Genetic Diversity of *Trypanosoma cruzi* in the United States of America: The Least Endemic Country for Chagas Disease

**DOI:** 10.3390/life14070901

**Published:** 2024-07-19

**Authors:** Arnau Llovera, Alba Abras, Anna Fernández-Arévalo, Cristina Ballart, Sandra Heras, Carmen Muñoz, Montserrat Gállego

**Affiliations:** 1 Independent Researcher, 17003 Girona, Spain; lloverarnau@gmail.com; 2 Laboratori d’Ictiologia Genètica, Departament de Biologia, Universitat de Girona, 17003 Girona, Spain; sandra.heras@udg.edu; 3 Secció de Parasitologia, Departament de Biologia, Sanitat i Medi Ambient, Facultat de Farmàcia i Ciències de l’Alimentació, Universitat de Barcelona, 08028 Barcelona, Spain; annaferar@gmail.com (A.F.-A.); cristinaballartferrer@ub.edu (C.B.); mgallego@ub.edu (M.G.); 4 Institut de Salut Global de Barcelona (ISGlobal), Hospital Clínic, Universitat de Barcelona, 08036 Barcelona, Spain; 5 Servei de Microbiologia, Hospital de la Santa Creu i Sant Pau, 08025 Barcelona, Spain; cmunoz@santpau.cat; 6 Institut de Recerca Biomèdica Sant Pau, 08041 Barcelona, Spain; 7 Departament de Genètica i Microbiologia, Universitat Autònoma de Barcelona, 08193 Barcelona, Spain; 8 CIBERINFEC (Centro de Investigación Biomédica en Red de Enfermedades Infecciosas), Instituto de Salud Carlos III, 28029 Madrid, Spain

**Keywords:** *Trypanosoma cruzi*, discrete typing unit, DTU, endemic country, genetic diversity, molecular epidemiology, mammalian hosts, triatomine vectors

## Abstract

Chagas disease (CD), caused by *Trypanosoma cruzi* and endemic in Latin America, has become an emergent health problem in non-endemic countries due to human migration. The United States (US) is the non-Latin American country with the highest CD burden and cannot be considered as non-endemic, since triatomine vectors and reservoir animals have been found. Populations of *T. cruzi* are divided into genetic subdivisions, which are known as discrete typing units (DTUs): TcI to TcVI and TcBat. Autochthonous human *T. cruzi* infection in the US is sporadic, but it may change due to environmental factors affecting the geographic distribution of triatomines. We aimed to perform a literature review of the genetic diversity of *T. cruzi* in triatomine vectors and mammalian hosts, including human cases, in the US. The 34 analyzed studies revealed the presence of *T. cruzi* in 18 states, which was mainly concentrated in Texas, Louisiana and New Mexico. TcI and TcIV were the principal DTUs identified, being TcI the most genotyped (42.4%; 917/2164). This study represents a first attempt to compile the molecular epidemiology of *T. cruzi* in the US, which is fundamental for predicting the progression of the infection in the country and could be of great help in its future management.

## 1. Introduction

Chagas disease (CD), also known as American trypanosomiasis, is a parasitic zoonosis caused by the protozoan *Trypanosoma cruzi* (Kinetoplastea: Trypanosomatidae). Initially, the disease was restricted to poor rural areas of Latin America (LA), where it is mainly transmitted by hematophagous triatomine vectors (Hemiptera: Reduviidae), commonly known as kissing bugs, that feed on mammalian blood and release infective forms of the parasite with their feces [1]. *Trypanosoma cruzi* can infect many of the 148 described triatomine species and almost all tissues of more than 180 mammalian species, contributing to the maintenance of the parasite in nature through the interchange of three distinct and interrelated cycles: wild or sylvatic (enzootic), peridomestic, and domestic [2,3,4,5].

Chagas disease began accidentally when humans invaded the wild cycle of *T. cruzi* and became infected. Since then, the transmission of the parasite evolved from enzootic to anthropozoonotic, being now well established among reservoir mammalian species, vectors, and human beings [6,7]. Although all triatomine species present in the Americas are considered potential vectors of *T. cruzi*, about 20 species of the genera *Triatoma*, *Rhodnius* and *Panstrongylus* are the most epidemiologically relevant in the transmission to humans [5,8]. Within the vectorial transmission of *T. cruzi*, there is an alternative pathway known as oral or foodborne, which takes place through the ingestion of beverages and food contaminated with feces of infected triatomines or secretions of parasite reservoirs as well as the consumption of raw/uncooked meat or blood from reservoirs [9]. Transmission can also occur by non-vectorial routes, i.e., blood transfusion, organ, and tissue transplantation, from mother to child during pregnancy or delivery (congenital route) and laboratory accidents [1].

Chagas disease has two stages. The acute phase lasts four to eight weeks and in 90–95% of cases evolves asymptomatically or with generally mild nonspecific signs. Oral transmission is related to more severe symptomatology [9]. The acute phase usually resolves spontaneously with a substantially decrease in parasitemia after approximately 90 days [10]. Congenital CD is considered an acute infection in the newborn and has mortality rates of around 2% [11]. After the acute period, untreated immunocompetent individuals enter the chronic phase, and most of them settle into a chronic asymptomatic stage. One to three decades post-acute infection, 30–40% of *T. cruzi*-infected patients will lead to symptomatic chronic disease with organ involvement associated with severe cardiac and gastrointestinal disorders [10,11]. The chronic phase is characterized by low and intermittent parasitemia [12].

Today, there are an estimated 6 to 8 million people with CD in the 21 continental LA countries considered endemic for the disease (within the vector distribution area), with 38,000 new cases and 12,000 deaths per year, and about 65 million people at risk of infection [13]. In the last few decades, CD has emerged as a global-scale issue resulting from human flows that have expanded the infection from rural to urban environments and across LA borders to reach non-endemic countries, especially in North America, Europe, and the Western Pacific region [14,15]. With nearly 20 million residents from CD endemic countries, the US is the leading recipient country for LA migrants as well as the non-Latin American country with the highest CD burden [16,17]. Even though most people living with CD in the US came from strictly endemic settings, the country cannot be labeled as non-endemic because there is evidence of established enzootic cycles of *T. cruzi* in the southern states, including several triatomines and mammalian species, and sporadic cases of autochthonous CD have also been described [3,18]. Therefore, given the particular and ambiguous position of the US in terms of endemicity, it could be roughly termed as the least endemic country for CD.

Regarding genetics, *T. cruzi* has a marked diversity. Taxonomic studies attempt to identify associations between the intraspecific diversity of the parasite and the clinical presentation of CD [19]. Although certain contributions of the different genotypes to clinical outcomes are intuited, the direct implications of the biological subdivisions of *T. cruzi* with the various manifestations of CD, as well as the distinct infectivity and virulence rates, have not been yet defined [20,21]. This is probably because human infection is a recent event in the evolutionary history of the parasite [20]. In LA endemic regions, some important clinical aspects have already been broadly defined as well as the type of transmission cycle and the geographic distribution linked to *T. cruzi* genotypes [21]. In the case of the US, *T. cruzi* transmission is spreading in the south of the country but is apparently restricted to the wild cycle with sporadic, but increasing, transmission to humans [3,22]. All in all, knowledge about the parasite genetic variability and its distribution in the territory could be of great help considering that the climate change may play a key role in the spread of *T. cruzi* vectors to the northern states and that they are increasingly tolerant to urbanized environments [12,23,24].

In this framework, the aim of this article was to perform a narrative review of the genetic diversity of *T. cruzi* in both triatomine vector species and mammalian hosts, including human cases, in the US. It will allow us to describe the current situation of the infection in the US to understand its evolution up to the present day as well as to predict its future steps. The information gathered will be directly applicable to CD management plans and will be useful for its control.

## 2. Chagas Disease Epidemiology in the US

According to estimates, there are 326,000 to 347,000 people with CD living in the US [17]. The states with the highest CD burden are California, Texas, Florida, and New York, with more than 10,000 reported cases, excluding undocumented immigrants [25]. Despite that fact, the level of underdiagnosis is extremely high (~99%) [26]. Most cases of CD reported in the US correspond to individuals infected in their countries of origin (imported CD). Women of childbearing age are a group of particular concern because approximately 40,000 women of reproductive age that are living in the US have chronic CD [27]. Therefore, assuming a 1% to 5% transmission rate, 400 to 2000 newborns with congenital CD can be estimated.

### Autochthonous Human Chagas Disease in the US

Autochthonous human infections due to vector-borne transmission remain rare in the US, but the fact that at least 11 species of triatomines have been recorded in the country cannot be underestimated [3,18]. Lynn et al. [22] found 76 cases of contemporary suspected (47) or confirmed (29) locally acquired CD between the years 2000 and 2018. There was an increase in the detection of chronic CD cases from 2007, corresponding to the introduction of blood donor screening for *T. cruzi* infection in the US [28]. Afterwards, another autochthonous case diagnosed in 2018 was reported in Missouri [29] and eight additionally suspected (4) or confirmed (4) autochtonous *T. cruzi* infections were diagnosed from May 2019 to July 2020 via the US blood donor screening system [30]. Previously, five more cases had already been identified, the first of which was notified in 1955 ([18] and references therein).

Taking all these data into account, at least 90 cases of autochthonously acquired CD are estimated to date in the US. Texas is by far the state with the highest number of reported cases of autochthonous CD [22,31]. However, probable or confirmed autochthonous CD cases have been reported in the states of Arizona, Arkansas, California, Louisiana, Mississippi, Missouri, and Tennessee [29,32,33].

## 3. *Trypanosoma cruzi* Genetic Diversity

The genetic structure of the *T. cruzi* population has long been considered predominantly clonal because of mostly asexual reproduction by binary fission coupled with occasional genetic exchange [34]. However, the clonal paradigm has been strongly challenged by the reported evidence of frequent recombination by sexual reproduction in the natural populations, which fits with the genetic heterogeneity of the parasite [35,36].

After many unsuccessful attempts to classify the intraspecific taxonomic structure of *T. cruzi* under alternative nomenclatures, the concept of discrete typing unit (DTU) was first defined in 1998 by Tibayrenc [37] as “sets of stocks that are genetically more related to each other than to any other stock and are identifiable by common molecular markers that act as tags”. Strains within DTUs are not identical clones but groups of interrelated clones that share profiles for a specific panel of markers, so these stocks could be further differentiated using additional markers due to accumulations of discrete mutations and events of genetic exchange [21,38].

The first consensus of *T. cruzi* intraspecific nomenclature was not reached until 1999 when the DTUs were divided into two groups named *T. cruzi* I (TcI) and TcII during the Satellite Meeting [39]. Afterwards, TcII was subdivided into five more units termed TcIIa–TcIIe [40,41] which were finally renamed in 2009, together with TcI, with the current nomenclature of six DTUs (TcI to TcVI) as recommended at the Second Satellite Meeting [42] (Table 1). In this same meeting of experts, TcV and TcVI were recognized as hybrid DTUs probably derived from TcII and TcIII. As for the remaining DTUs, TcI and TcII are considered pure ancestral lineages with long-standing independent evolution, while the origin of TcIII and TcIV is still under debate [21,43].

However, the second revision of *T. cruzi* nomenclature did not include subdivisions for TcI due to the need to complete studies based on additional markers [44]. In 2010, Cura et al. [45] reported five genotypes within TcI, termed TcIa–TcIe, through the characterization of the intergenic region of the mini-exon gene (SL-IR). Genotype TcIe was detected for the first time, whereas TcIa-d had been previously described ([44] and references therein). In addition, a seventh new DTU, called TcBat, closest to TcI and associated with bats was first reported in Brazil by Marcili et al. [46]. To our knowledge, only one case of human *T. cruzi* infection attributed to the TcBat genotype has been described [47].

DTUs have differential geographical distribution in endemic regions and transmission cycles. In brief, TcI is the most widely distributed DTU in LA and is present in both domestic and sylvatic cycles. TcII, TcV, and TcVI are predominant in domestic cycles and circulate mainly in the Southern Cone. TcIII and TcIV are predominant in sylvatic cycles and are principally found in the Amazon region [21].

## 4. *Trypanosoma cruzi* DTUs in the US

**Figure 1:**
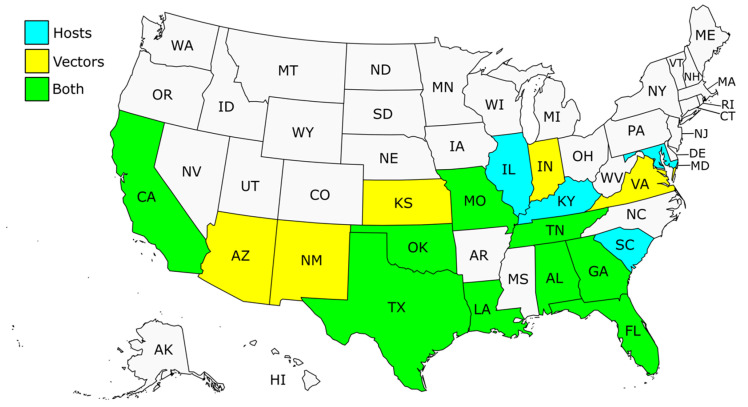
States of US with *T. cruzi* DTUs genotyped in mammalian hosts (blue), triatomine vectors (yellow) or both (green). In the case of the state of Missouri (MO), Curtis-Robles et al. [75] reported a triatomine (*Triatoma sanguisuga*) resulted positive by PCR for *T. cruzi* DNA, but it was not possible to type the DTU. The outline map was taken from https://simplemaps.com/resources/svg-maps, accessed on 20 June 2024. AL, Alabama; AK, Alaska; AZ, Arizona; AR, Arkansas; CA, California; CO, Colorado; CT, Connecticut; DE, Delaware; FL, Florida; GA, Georgia; HI, Hawaii; ID, Idaho; IL, Illinois; IN, Indiana; IA, Iowa; KS, Kansas; KY, Kentucky; LA, Louisiana; ME, Maine; MD, Maryland; MA, Massachusetts; MI, Michigan; MN, Minnesota; MS, Mississippi; MO, Missouri; MT, Montana; NE, Nebraska; NV, Nevada; NH, New Hampshire; NJ, New Jersey; NM, New Mexico; NY, New York; NC, North Carolina; ND, North Dakota; OH, Ohio; OK, Oklahoma; OR, Oregon; PA, Pennsylvania; RI, Rhode Island; SC, South Carolina; SD, South Dakota; TN, Tennessee; TX, Texas; UT, Utah; VT, Vermont; VA, Virginia; WA, Washington; WI, Wisconsin; WY, Wyoming.

In this review, a literature search was conducted in the scientific databases Web of Science, Scopus and Pubmed between September 2023 and March 2024 without time or language restrictions to identify relevant publications on the topic. The combination of search terms used to find potential studies was (*Trypanosoma cruzi* OR Chagas disease) AND (discrete typing unit OR DTU OR genotype OR lineage OR genetic diversity OR genetic variability) AND (United States OR US OR USA). In addition, the references of the retrieved articles were examined for other important publications that might have been missed. Accordingly, we have compiled information on the genetic characterization of *T. cruzi* in the US from a total of 34 studies that reported the genetic diversity of the parasite by DTUs in mammalian hosts, including humans, and triatomine vectors in different states of the country [31,32,45,48,49,50,51,52,53,54,55,56,57,58,59,60,61,62,63,64,65,66,67,68,69,70,71,72,73,74,75,76,77,78] (Appendix A: Table A1 and Table A2). Based on the gathered data from these typing studies in the US, 18 states of the country have reported *T. cruzi* DTUs in mammalian hosts, triatomine vectors or both (Figure 1). Typing protocols and caveats are further described in Section 6.

**Figure 2:**
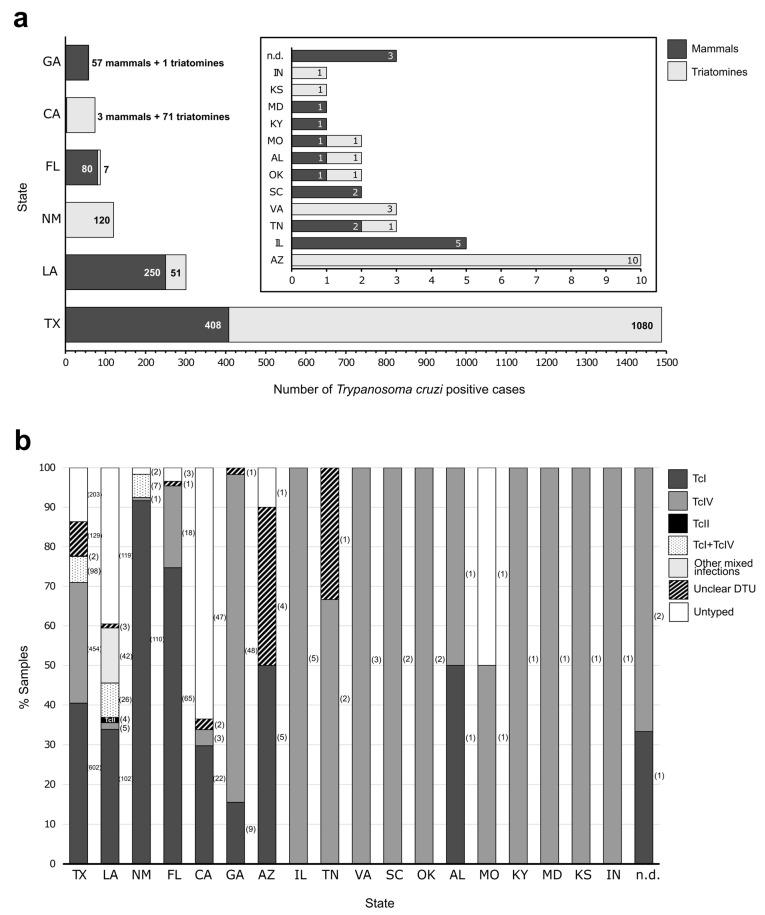
Trypanosoma cruzi DTUs identification according to the states of the US. (**a**) Number of cases positive for *T. cruzi* in mammalian hosts (including humans) and triatomine vectors (n hosts = 815; n vectors = 1349; global n = 2164). States with up to 10 *T. cruzi* positive cases are shown with an adapted scale in the box at the top of the figure. (**b**) Percentage of samples typed according to the states of the US. The number of types per state is bracketed. Other mixed infections include TcI + TcII, TcI + TcVI, TcI + TcII + TcVI, TcI + TcII/V, TcI + TcII/V/VI, TcI + TcIV + TcII/V, TcI + TcII + TcV + TcVI, TcI + TcII + TcIV + TcV + TcVI, TcII + TcIV and TcII + TcVI; Unclear DTU, it was not possible to genotype at the level of a single DTU. AL, Alabama; AZ, Arizona; CA, California; FL, Florida; GA, Georgia; IL, Illinois; IN, Indiana; KS, Kansas; KY, Kentucky; LA, Louisiana; MD, Maryland; MO, Missouri; NM, New Mexico; OK, Oklahoma; SC, South Carolina; TN, Tennessee; TX, Texas; VA, Virginia; n.d., state of origin not determined.

The global molecular prevalence, i.e., the number of samples with evidence of *T. cruzi* DNA by PCR or blood culture over the total number of samples tested, was 25.6% (2164/8460) (Table A1 and Table A2). However, it should be noted that this calculation is not precise, since several studies started from samples previously selected as positive [32,63,71,78]. Once excluded, the prevalence drops to 24%. Of these positive specimens, 62.3% were found in triatomine vectors. The vast majority of *T. cruzi* positives were concentrated in six states: Texas, Louisiana, New Mexico, Florida, California and Georgia, all of them located in the southern part of the US (Figure 2a). The remaining 12 states reached up to 10 cases, and there were also two *T. cruzi* positive domestic dogs and one Rhesus macaque reported by Roellig et al. [32] for which the state of origin could not be determined. Indeed, the state of Texas alone accounted for almost 50% of the cases. DTUs TcI and TcIV were the principal lineages identified in the country (Figure 2b), being TcI the most genotyped DTU (42.4%; 917/2164). It was present in eight states and was the predominant DTU in Texas, New Mexico, Florida and Arizona. TcIV was the second most present DTU (25.5%; 551/2164), with records in all the states listed except Arizona. Mixed infections TcI + TcIV were also reported in the 6.1% of the samples included in this study. TcII was only found in Louisiana [49,70]. Finally, the fact that 517 samples (23.9%) could not be characterized cannot be underestimated. Of them, in 141 cases, some DTUs could be ruled out, but the genotype could not be accurately determined. Factors that may have influenced the success of the characterization are discussed in Section 6.

### 4.1. Trypanosoma cruzi DTUs Identified in Hosts

**Figure 3:**
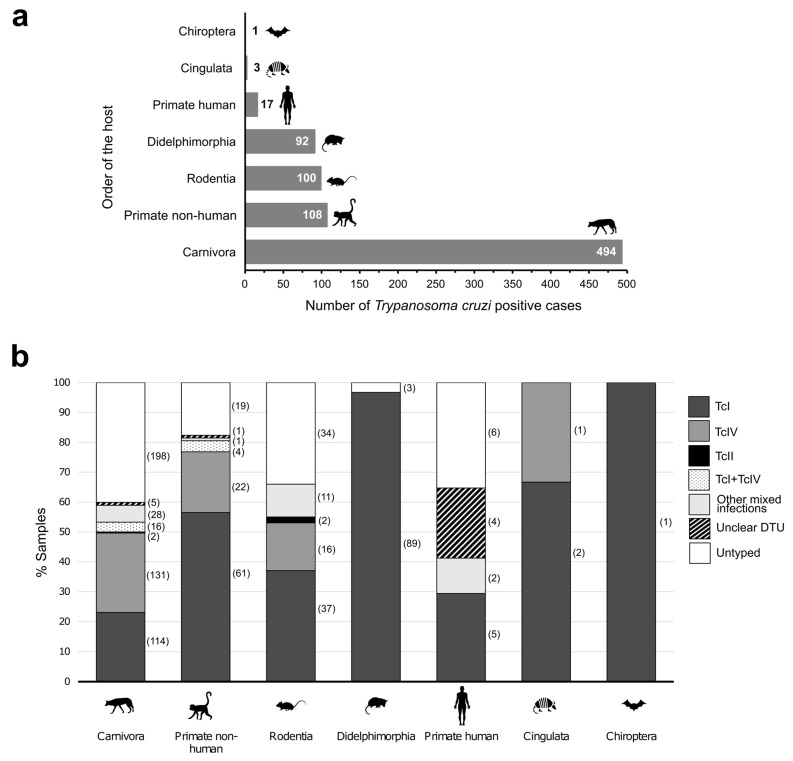
*Trypanosoma cruzi* DTUs identification in mammalian hosts. (**a**) Number of cases positive for *T. cruzi* according to the mammal host order (n = 815). (**b**) Percentage of samples typed according to the mammal host order. The number of types per order are bracketed. The distribution of species within each order is as follows: Chiroptera: evening bat (*Nycticeius humeralis*) (1); Cingulata: nine-band armadillo (*Dasypus novemcinctus*) (3); Didelphimorphia: Virginia opossum (*Didelphis virginiana*) (92); Rodentia: southern plains woodrat (*Neotoma micropus*) (36), hispid cotton rat (*Sigmodon hispidus*) (3), rock squirrel (*Otospermophilus variegatus*) (1), house mouse/cotton mouse (*Mus musculus* and *Peromyscus gossypinus*) (34), eastern woodrat (*Neotoma floridana*) (12), northern pygmy mouse (*Bayomis taylori*) (1), white-footed mouse (*Peromyscus leucopus*) (3), hispid pocket mouse (*Chaetodipus hispidus*) (1), Mexican spiny pocket mouse (*Liomys irroratus*) (1), house mouse (*Mus musculus*) (2), cotton mouse (*Peromyscus gossypinus*) (3), cactus mouse (*Peromyscus eremicus*) (1); spotted ground squirrel (*Xerospermophilus spilosoma*) (1), western harvest mouse (*Reithrodontomys megalotis*) (1); Primate non-human: ring-tailed lemur (*Lemur catta*) (3), rhesus macaque (*Macaca mulatta*) (42), pig-tailed macaque (*Macaca nemestrina*) (2), cynomolgus macaque (*Macaca fascicularis*) (59), and baboon (*Papio* spp.) (2); Carnivora: domestic dog (*Canis lupus familiaris*) (206), raccoon (*Procyon lotor*) (189), domestic cat (*Felis catus*) (80), coyote (*Canis latrans*) (11), striped skunk (*Mephitis mephitis*) (7), and gray fox (*Urocyon cinereoargenteus*) (1).

DTUs reported in mammals according to host species and the state of localization are summarized in Table A1. The states with more positive *T. cruzi* cases among mammals were Texas (408) and Louisiana (250) (Figure 2a). The molecular prevalence in mammalian hosts was 12.3%, excluding studies based on preselected samples [32,63,71]. Most of the samples positive by *T. cruzi* were obtained from Carnivora (60.6%; 494/815) and particularly from domestic dogs (206/494) (Figure 3). DTUs could not be typed for all samples positive for the presence of *T. cruzi* DNA. The typing success rate was 66.9% (545/815) with the highest number of uncharacterized samples from carnivores (41.1%; 203/494). In the case of humans, all positive isolates were from autochthonous US cases of *T. cruzi* infection, and the 70.6% (12/17) were characterized from whole blood [31]. There is no information about the sample source for the remaining five isolates [32].

Regarding *T. cruzi* typing in mammalian hosts, the most reported DTUs were TcI and TcIV (Figure 3b). TcI was the only genotype found in Didelphimorphia and in the single Chiroptera specimen. Indeed, TcI was present in all orders of mammalian hosts analyzed and was also the most common lineage found in all of them except for Carnivora, in which TcIV was the dominant one. In fact, this was to be expected, since TcI is the most ubiquitous DTU and is present in both domestic and sylvatic cycles [79]. TcIV was found in Carnivora, non-human primates, Rodentia and Cingulata. On the other hand, TcII was detected displaying single infection in two mice (Rodentia) and two racoons (Carnivora) from Louisiana [49,70]. Previously, Majeau et al. [80] had already described raccoons as a major reservoir of *T. cruzi* in Louisiana with a reported molecular prevalence of 33.6% in two metropolitan areas in this state. The most reported mixed infection involved TcI and TcIV, but other DTU combinations were also detected, including the remaining DTUs except TcBat.

### 4.2. Trypanosoma cruzi DTUs Identified in Vectors

**Figure 4:**
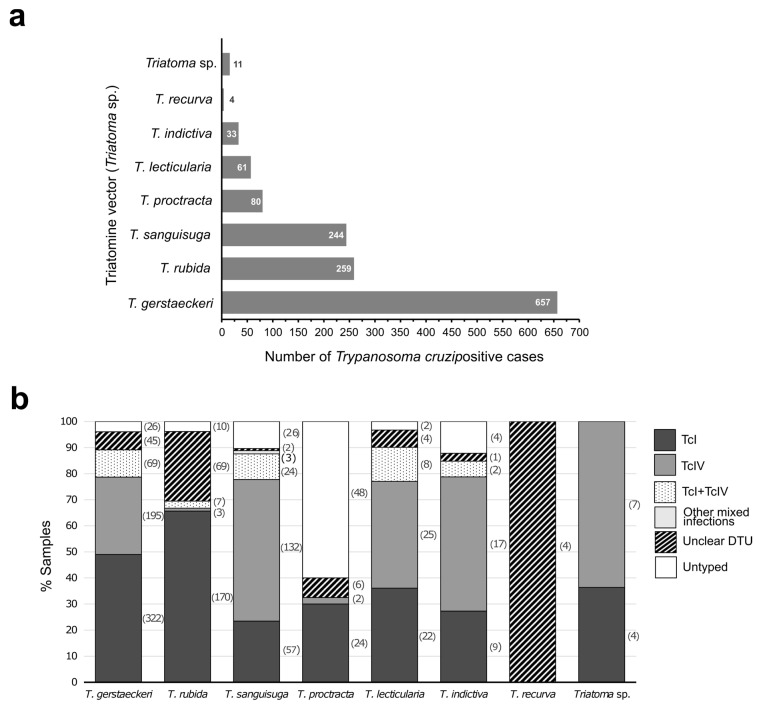
*Trypanosoma cruzi* DTUs identification in triatomine vectors. (**a**) Number of cases positive for *T. cruzi* according to the triatomine species (n = 1349). (**b**) Percentage of samples typed according to the triatomine species. The number of types per species are bracketed.

Eleven species of triatomine kissing bugs are found in 27 states distributed across the lower two thirds of the US: *Triatoma protracta*, *T. sanguisuga*, *T. lecticularia*, *T. rubida*, *T. gerstaeckeri*, *Paratriatoma hirsuta*, *T. indictiva*, *T. neotomae*, *T. rubrofasciata*, *T. recurva*, and *T. incrassata* [81]. In this review, seven species of *Triatoma* were reported as positive for *T. cruzi* (Figure 4). Furthermore, 11 triatomines reported by Curtis-Robles et al. [75] could not be assigned to a specific species. The states with more positive *T. cruzi* cases among vectors were Texas (1080) and New Mexico (120) (Figure 2). *Trypanosoma cruzi* DTUs reported in vectors according to triatomine species and the state of localization are listed in Table A2.

The overall molecular prevalence in triatomine vectors was 50.5%, excluding studies based on preselected samples [32,78]. The triatomine species with more reported *T. cruzi*-positive cases was *T. gerstaeckeri*, which was followed by *T. rubida* and *T. sanguisuga* (Figure 4a). *Triatoma gerstaeckeri* has a limited range but is common in Texas, which explains the fact that it is the most collected species [18]. TcI and TcIV were reported in all the triatomine species (Figure 4b). In the case of the four *T. recurva* with *T. cruzi* collected by Flores-López et al. [78], the unclear DTU refers to TcI or TcIV. The most common DTU was TcI (45.1%; 608/1349) followed by TcIV (28.2%; 381/1349). Overall, 18.3% of the *T. cruzi* positive triatomines (247/1349) could not be typed (116) or had an unclear DTU (131). The presence of the rest of the DTUs was extremely low. Hwang et al. [72] found that two *T. protracta* isolates from California were related to TcII and TcVI groups. On the other hand, Dumonteil et al. [77] detected for the first time the presence of TcII/TcV in triatomines in the US. These genotypes were found as part of mixed infections with TcI (2) and TcI + TcIV (1), and the authors were unable to distinguish them at the level of a single DTU.

## 5. The Importance of Identifying DTUs in the US

Taxonomic studies have attempted to identify associations between DTUs and the clinical presentation of CD and clarify the geographic distribution of *T. cruzi* genotypes in endemic regions and transmission cycles [19]. The description of the contribution of each *T. cruzi* DTU to the forms of chronic CD is far from clear and is further hampered by the fact that the pathophysiology of the disease is not only determined by the genetic diversity of the parasite but also by complex host–pathogen interactions (with multiple unknown factors) as well as environmental factors [21,82]. Another point to note is that the full extent of intra-lineage diversity of *T. cruzi* in the progression of CD has not yet been elucidated. Probably, to identify clear associations between parasite diversity and the disease outcomes, DTUs but also intra-DTU sublevels must be considered [70,83]. Indeed, due to its great intra-diversity, five genotypes within TcI have already been described [44,45]. However, based on the premise that infection with specific strains leads to distinct outcomes, there are a growing number of studies trying to investigate the association of *T. cruzi* DTUs with different outcomes of CD [82,84]. From the information gathered in this type of studies that assign DTUs to *T. cruzi* populations, it has been possible to relate the genetic diversity of the parasite with its ecoepidemiological features and the presentation of CD in humans [85]. Except for TcBat, of which only one human case has been described in Colombia [47], and TcIII, which is rare in humans, the remaining DTUs have been associated with clinical manifestations of chronic CD. TcI and TcIV have been linked mainly to cardiac disorders, while TcII, TcV and TcVI have also been connected to digestive outcomes [85]. In addition, TcV has also been related to an increased risk of congenital infection [86].

On the other hand, climate change is an emerging important factor contributing to the epidemiology of CD in general terms but with a particularly important impact in the US due to the endemic condition of the southern part of the country [87]. Consequently, the distribution range of triatomines may expand northward, leading to an increased risk of autochthonous cases of CD in humans, even in areas that are not currently endemic for the infection [23,81]. Studies examining circulating DTUs in the country will help to predict the potential geographic distribution of *T. cruzi* genotypes in new environmental scenarios. Other factors that may contribute to changes in the epidemiology of CD in the US include increasing migration, rapid population growth, rising urbanization, and growing poverty [87,88].

Regarding treatment, current drugs for treating CD are benznidazol (BZ) and nifurtimox (NF). Both are available from the 1970s, require prolonged treatment and cause severe side effects [89]. It is well documented that treatment should be offered the earlier the better, with proven high efficacy in acute and congenital cases and improved clinical results in the chronic indeterminate phase [90]. Nevertheless, BZ and NF show high variable efficacy, with reported differences in treatment outcomes in distinct geographic areas, as well as in murine models inoculated with different *T. cruzi* DTUs ([89] and references therein). Although it is not possible to directly connect each DTU to a specific treatment behavior, a variation in pathogenicity and susceptibility to treatment of *T. cruzi* genotypes is intuited [21,91]. For instance, Vela et al. [92] observed a lower susceptibility of TcI at the trypomastigote stage to BZ. Revollo et al. [93] also reported TcI trypomastigotes as more resistant to BZ and NF, although some TcII and TcV strains behaved similarly. A factor to consider is that susceptibility to treatment may vary within the same DTU and at different stages of the parasite life cycle (i.e., trypomastigote, epimastigote and amastigote) [89,93]. Therefore, knowledge of the identity of *T. cruzi* DTUs circulating in the US is important to establish possible connections to human infections, to detect differences in lineage behavior to current treatment, and to design and develop new potential therapeutic targets [78].

In essence, understanding the genetic heterogeneity of circulating *T. cruzi* populations in human hosts, sylvatic and domestic reservoirs and vectors from a comprehensive perspective will be of particular interest to help manage CD in the country [94].

## 6. The Problem of Identifying DTUs

Molecular tools are the most used to genotype *T. cruzi* DTUs in humans, reservoirs and vectors. The most common markers for *T. cruzi* typing are the 24sα rRNA gene, the mitochondrial cytochrome oxidase subunit 2 (COII) gene, the mini-exon gene spliced leader intergenic region (SL-IR), and the 18S rRNA gene, among others (Table A1 and Table A2). In addition to the variety of markers, there is a wide range of algorithms and characterization techniques available, which adds even more complexity to the interpretation of the results [95,96,97].

One of the main problems of direct typing in biological samples is the lack of sensitivity due to the single or low copy number of the DNA markers used [98,99,100]. Low and fluctuant parasitemia, typical of the chronic phase of CD, is also another drawback to achieving good accuracy in genotyping [101]. Precisely because they are sometimes not sensitive enough to be used in biological samples, typing schemes are in occasions applied to cultured stocks of the parasite [96]. These kinds of studies involving parasite amplification, either by in vitro culture or by passages in experimental models, may bias the *T. cruzi* populations [20,100].

Highly repetitive sequences used for the molecular diagnosis of CD, such as satellite DNA (SatDNA) and the minicircle hypervariable region of the kinetoplastic DNA (kDNA), have been proposed as an improved option to increase sensitivity in *in vivo* typing studies [98,99]. Both sequences are represented in around 10^5^ copies per parasite genome and are therefore easily detectable by molecular techniques [99,102,103]. The main limitation is that the analysis of the signature patterns of each DTU in these highly repetitive markers implies sequencing. In the case of SatDNA, the main limitation is that the proposed approach is not yet capable of distinguishing between the presence of hybrid lineages (TcV and TcVI) and the existence of mixed infections with TcI or TcIII and TcII [98]. More recently, serological typing or serotyping, based on polymorphic antigens that detect strain-specific antibody signatures, has also been suggested as an alternative method to molecular genotyping [100]. However, this approach still needs to be further explored to determine its application in the characterization of *T. cruzi* DTUs.

Another problem related to *T. cruzi* genotyping is the tissue tropism detected for different DTUs [95,104]. This phenomenon was first described by Macedo and Pena [105] as the “clonal-histotropic model of the pathogenesis of CD” and is also reported in non-human mammalian hosts [106,107]. Most studies that characterize *T. cruzi* populations from clinical samples are based on isolates recovered from peripheral blood [95]. It is important to note that blood isolates may not reveal all *T. cruzi* DTUs infecting a particular patient, as other genotypes may be circulating at low loads or being retained in tissues. Therefore, the mostly present DTU could be having a masking effect with respect to other genotypes [20].

The general advice could be that the accurate classification of *T. cruzi* DTUs requires the use of several markers, as characterization based on a single marker may lead to the assignment of an erroneous genotype to the isolate [94,105]. Indeed, for an optimal typing, multiple isolates from the patient should be analyzed, since different *T. cruzi* populations can simultaneously infect the same individual (mixed infections) and not be detected with only one sample [108].

## 7. Final Remarks

Although most reports of *T. cruzi* infections in mammals and triatomine vectors occurred in the southern US, new cases are progressively appearing in northern states, such as raccoons (*Procyon lotor*) with TcIV detected in Illinois and Missouri [57] (Table A1) and the *T. sanguisuga* also with TcIV in Indiana [75] (Table A2). As pointed out in previous sections, this pattern could increase because of climate change. So, epidemiological surveillance programs for *T. cruzi* infection in the US should be increased to cover and detect this eventual expansion of the number of cases in both hosts and vectors that can ultimately help to estimate risk to human health [70]. Texas accounts for most reported cases of *T. cruzi* infection in the country, which is probably influenced by the numerous studies conducted in this state due to its border condition with Mexico. Therefore, the study area should be expanded to be able to draw a true unbiased map of cases in the territory.

TcI has been the most reported DTU in the studies included in this review, being present in 37.9% (309/815) of the mammals analyzed and in 45.1% (608/1349) of the vectors. TcIV was the second most reported DTU, with 20.9% (170/815) of mammals and 28.2% (381/1349) of vectors. TcII, TcV and TcVI were also present in the country but to a lesser extent and mainly as part of mixed infections. These combinations of DTUs were almost entirely reported in the state of Louisiana (Figure 2b). In humans, TcI was reported in 29.4% (5/17) of *T. cruzi*-positive cases. In two more cases, TcI was found in combination with TcII/TcV/TcVI, although it was not possible to determine which genotype or genotypes were involved [31,32]. The same occurred in four other cases in which non-TcI was found. In the remaining six cases, the DTU could not be determined. It indicates the existence of several circulating DTUs, in addition to TcI, among autochthonous cases of human CD in the USA, which accentuates the need to determine the genotypes present in each human patient.

However, the totality of *T. cruzi* diversity present in the country is probably not being fully detected because of the low resolution of the characterization markers used, the lack of a typing reference, the bias produced by the main use of peripheral blood as the sample of choice and the low parasitemia in chronic infections. All these factors hamper *T. cruzi* typing and make the characterization of the parasite complex and laborious. Thus, new markers and technologies are needed to optimize the process and to identify diversity at the intra-DTU level [83,97].

On the other hand, this study also highlights the need for an increased *T. cruzi* characterization throughout the US to predict the possible future pathways of the infection in the country and to focus strategies to deal with CD. Currently, most cases of CD reported in the US correspond to individuals infected in their countries of origin, and autochthonous human *T. cruzi* infection occurs sporadically and because of enzootic transmission from sylvatic cycles maintained in animals. However, in such an unstable and changing environmental scenario, CD could become epidemiologically relevant in the country in the near future. In terms of human cases, TcI is the DTU of most concern, but the presence of TcIV in the country, as well as TcII, TcV and TcVI, may lead to potential autochthonous infections by these DTUs in the US. In addition, specific genotypes also appear to circulate in the US. Flores-López et al. [78] identified the so-called TcIV-USA in the southwestern US, which is described as a divergent American branch of TcIV (Table A2). Thus, it is crucial to study the molecular epidemiology of *T. cruzi* in the area and its progression in the different states of the country. This information may be of special interest in the development of future management and control plans for CD in the US, including screening and epidemiological surveillance protocols in humans but also interventions targeting mammalian *T. cruzi* hosts that act as a reservoir as well as triatomine vectors.

## Figures and Tables

**Table 1:** Nomenclature for *Trypanosoma cruzi* intraspecific genetic diversity.

Satellite Meeting [39]	Brisse et al. [40,41]	Zingales et al. [42]
TcI	TcI	TcI
TcII	TcIIa	TcIV
	TcIIb	TcII
	TcIIc	TcIII
	TcIId	TcV
	TcIIe	TcVI

## Data Availability

Not applicable.

## Appendix A

**Table A1:** *Trypanosoma cruzi* DTUs reported in mammalian hosts per state in the US.

Reference	Host	State	Target	Method	Molecular Prevalence ^a^ P/Tested (%)	Typed typ./P (%)	DTU (Number)			
							TcI	TcIV	Other	Mixed Infections
Roellig et al. [32]	Human (*Homo sapiens sapiens*)	CA	SL-IR; 24Sα; 18S	PCR; MLEE; RAPD; STR	2/2 (100)	2/2 (100)	2			
		TX			2/2 (100)	2/2 (100)	2			
		LA			1/1 (100)	1/1 (100)	1			
	Domestic dog (*Canis lupus familiaris*)	n.d.			2/2 (100)	2/2 (100)		2 *		
		TN			1/1 (100)	1/1 (100)			1 TcI/TcIV *	
		OK			1/1 (100)	1/1 (100)		1 *		
		SC			1/1 (100)	1/1 (100)		1 *		
		CA			1/1 (100)	1/1 (100)		1 *		
	Virginia opossum (*Didelphis virginiana*)	GA			6/6 (100)	6/6 (100)	6			
		FL			6/6 (100)	6/6 (100)	6			
		LA			2/2 (100)	2/2 (100)	2			
		AL			1/1 (100)	1/1 (100)	1			
	Raccoon (*Procyon lotor*)	GA			45/45 (100)	45/45 (100)	2	43 *		
		FL			16/16 (100)	16/16 (100)		15 *	1 TcI/TcIV *	
		SC			1/1 (100)	1/1 (100)		1 *		
		MD			1/1 (100)	1/1 (100)		1 *		
		TN			1/1 (100)	1/1 (100)		1 *		
	Ring-tailed lemur (*Lemur catta*)	GA			3/3 (100)	3/3 (100)		3 *		
	Rhesus macaque (*Macaca mulatta*)	n.d.			1/1 (100)	1/1 (100)	1			
		GA			1/1 (100)	1/1 (100)			1 TcI/TcIV *	
	Nine-band armadillo (*Dasypus novemcinctus*)	GA			1/1 (100)	1/1 (100)		1 *		
		LA			2/2 (100)	2/2 (100)	2			
	Striped skunk (*Mephitis mephitis*)	GA			1/1 (100)	1/1 (100)		1 *		
Charles et al. [48]	Southern plains woodrat (*Neotoma micropus*)	TX	24Sα	PCR; sequencing	35/104 (34)	23/35 (66)	10	13		
	Striped skunks (*M. mephitis*)	TX			4/4 (100)	4/4 (100)	1	3		
	Racoon (*P. lotor*)	TX			12/20 (60)	5/12 (42)		5		
	Hispid cotton rat (*Sigmodon hispidus*)	TX			2/2 (100)	2/2 (100)		2		
	Rock squirrel (*Otospermophilus variegatus*)	TX			1/1 (100)	1/1 (100)		1		
Herrera et al. [49]	House mouse (*Mus musculus*); Cotton mouse (*Peromyscus gossypinus*)	LA	SL-IR; 24Sα; 18S	PCR; sequencing	34/44 (77)	20/34 (59)	16		2 TcII	1 TcI + TcII 1TcII + TcIV
	Eastern woodrat (*Neotoma floridana*)	LA			11/15 (73)	3/11 (27)	2			1 TcII + TcIV
Hodo et al. [50]	Evening bat (*Nycticeius humeralis*)	TX	SL-IR; 24Sα; 18S; COII	MTq-PCR	1/593 (0.2)	1/1 (100)	1			
Curtis-Robles et al. [51]	Racoon (*P. lotor*)	TX	TcSC5D	Sequencing	49/70 (70)	11/49 (22)		10		1 TcI + TcIV
Aleman et al. [52]	Northern pygmy mouse (*Bayomis taylori*)	TX	18S	Sequencing	1/4 (25)	1/1 (100)	1			
	Southern plains woodrat (*N. micropus*)	TX			1/5 (20)	1/1 (100)	1			
	White-footed mouse (*Peromyscus leucopus*)	TX			3/87 (3)	3/3 (100)	3			
	Hispid cotton rat (*Sigmodon hispidus*)	TX			1/13 (8)	1/1 (100)	1			
	Hispid pocket mouse (*Chaetodipus hispidus*)	TX			1/2 (50)	1/1 (100)	1			
	Mexican spiny pocket mouse (*Liomys irroratus*)	TX			1/44 (2)	1/1 (100)	1			
Curtis-Robles et al. [53]	Domestic dog (*C. lupus familiaris*)	TX	SL-IR; TcSC5D	MTq-PCR; sequencing	15/86 (17)	15/15 (100)	9	5		1 TcI + TcIV
Curtis-Robles et al. [54]	Domestic dog (*C. lupus familiaris*)	TX	SL-IR	MTq-PCR	5/184 (3)	4/5 (80)	4			
Garcia et al. [31]	Human (*H. sapiens sapiens*)	TX	SL-IR; 24Sα; 18S	PCR; sequencing	12/15 (80)	6/12 (50)			4 TcII/V/VI	2 TcI + TcII/V/VI
Meyers et al. [55]	Domestic dog (*C. lupus familiaris*)	TX	SL-IR	MTq-PCR	3/528 (0.6)	2/3 (67)		1		1 TcI + TcIV
Hodo et al. [56]	Rhesus macaque (*M. mulatta*)	TX	SL-IR; 24Sα; 18S; COII	MTq-PCR	33/41 (80)	33/33 (100)	18	13		2 TcI + TcIV
	Virginia opossum (*D. virginiana*)	TX			4/5 (80)	4/4 (100)	4			
	Racoon (*P. lotor*)	TX			2/5 (40)	2/2 (100)		2		
	Striped skunk (*M. mephitis*)	TX			2/3 (67)	2/2 (100)	1	1		
Vandermark et al. [57]	Racoon (*P. lotor*)	IL	SL-IR; 24Sα	PCR; sequencing	5 (37 global)	5/5 (100)		5		
	Racoon (*P. lotor*)	KY			1 (37 global)	1/1 (100)		1		
	Racoon (*P. lotor*)	MO			1 (37 global)	1/1 (100)		1		
Hodo et al. [58]	Domestic dog (*C. lupus familiaris*)	TX	SL-IR	MTq-PCR	53/559 (9)	6/53 (11)	5	1		
Herrera et al. [59]	Rhesus macaque (*M. mulatta*)	LA	SL-IR	NGS; MB	7/8 (88)	7/7 (100)	5			1 TcI + TcIV 1 TcI + TcVI
	Pig-tailed macaque (*Macaca nemestrina*)	LA			2/2 (100)	2/2 (100)	2			
	Baboon (*Papio* spp.)	LA			2/2 (100)	2/2 (100)	2			
Dumonteil et al. [60]	Domestic dog (*C. lupus familiaris*)	LA	SL-IR	PCR; NGS; MB	73/540 (14)	40/73 (55)	25			10 TcI + TcIV 2 TcI + II + V + VI 2 TcI + II + IV + V + VI 1 TcI + TcII
Hodo et al. [61]	Coyote (*Canis latrans*)	TX	SL-IR; 24Sα; 18S; COII	MTq-PCR	10/120 (8)	10/10 (100)	10			
	Racoon (*P. lotor*)	TX			15/24 (62)	15/15 (100)		15		
Meyers et al. [62]	Domestic dog (*C. lupus familiaris*)	TX	SL-IR	MTq-PCR	4/1610 (0.2)	3/4 (75)		2		1 TcI + TcIV
Pronovost et al. [63]	House mouse (*M. musculus*)	LA	SL-IR	NGS; MB	2/2 (100)	2/2 (100)				1 TcI + II + VI 1 TcII + VI
	Cotton mouse (*P. gossypinus*)	LA			3/3 (100)	3/3 (100)				1 TcII + TcVI 1 TcI + II + VI 1 TcI + II + V + VI
	Eastern woodrat (*N. floridana*)	LA			1/1 (100)	1/1 (100)				1 TcI + II + IV + VI
Zecca et al. [64]	Domestic cat (*Felis catus*)	TX	SL-IR; 24Sα; 18S; COII	MTq-PCR	3/167 (2)	3/3 (100)	3			
Zecca et al. [65]	Virginia opossum (*D. virginiana*)	TX	SL-IR	MTq-PCR	15/100 (15)	15/15 (100)	15			
Dumonteil et al. [66]	Domestic cat (*Felis catus*)	LA	SL-IR	PCR; sequencing	70/284 (25)	19/70 (27)			3 **	16 **
Rodríguez et al. [67]	Domestic dog (*C. lupus familiaris*)	TX	SL-IR; 24Sα;	PCR	43/95 (45)	40/43 (93)	30	9		1 TcI + TcIV
	Domestic cat (*Felis catus*)	TX			7/24 (42)	3/7 (43)	2	1		
	Cactus mouse (*Peromyscus eremicus*)	TX			1/1 (100)	1/1 (100)				1 TcI + TcIV
	Spotted ground squirrel (*Xerospermophilus spilosoma*)	TX			1/1 (100)	1/1 (100)				1 TcI + TcIV
	Western harvest mouse (*Reithrodontomys megalotis*)	TX			1/1 (100)	1/1 (100)	1			
	Gray fox (*Urocyon cinereoargenteus*)	TX			1/1 (100)	1/1 (100)	1			
	Coyote (*C. latrans*)	TX			1/1 (100)	1/1 (100)	1			
Padilla et al. [68]	Cynomolgus macaque (*Macaca fascicularis*)	TX	LSU; SL-IR	PCR	59/64 (92)	40/59 (68)	33	6		1 TcI + TcIV
Torhorst et al. [69]	Virginia opossum (*D. virginiana*)	FL	SL-IR; 24Sα; 18S; COII	MTq-PCR	58/112 (52)	55/58 (95)	55			
Majeau et al. [70]	Racoon (*P. lotor*)	LA	SL-IR	NGS	40/119 (34)	29/40 (73)	19		2 TcII	1 TcI + TcIV 7 ***
Landsgaard et al. [71]	Domestic dog (*C. lupus familiaris*)	TX	SL-IR	PCR	4/4 (100)	3/4 (75)	1	2		

CA, California; TX, Texas; LA, Louisiana; n.d., not determined; TN, Tennessee; OK, Oklahoma; SC, South Carolina; GA, Georgia; FL, Florida; AL, Alabama; MD, Maryland; IL, Illinois; KY, Kentucky; MO, Missouri; MLEE, multilocus enzyme electrophoresis; RAPD, random amplified polymorphic DNA; STR, short tandem repeats (microsatellites); NGS, next-generation sequencing; MB, metabarcoding; MTq-PCR, Multiplex TaqMan Real-Time PCR; typ., typed; SL-IR, spliced leader intergenic region of the mini-exon; 18S, 18S ribosomal DNA; 24Sα, 24Sα ribosomal DNA; COII, cytochrome oxidase II; LSU, large subunit rRNA; TcSC5D, TcSC5D gene. ^a^ Molecular prevalence was calculated as the number of samples with evidence of *T. cruzi* DNA by means of PCR or hemoculture (positive, P) with respect to the total number of samples analyzed (tested). Molecular prevalences in Roellig et al. [32], Pronovost et al. [63], and Landsgaard et al. [71] do not match reality since positive samples were pre-selected for the study. * TcIV was typified as TcIIa according to the previous nomenclature (see Table 1). ** Cats were predominantly infected with parasites from TcI and TcVI DTUs, and to a lesser extent from TcIV and TcV DTUs. *** Mixed infections of TcI, TcII, TcIV, TcV and TcVI in different combinations and proportions.

**Table A2:** *Trypanosoma cruzi* DTUs reported in triatomine vectors per state in the US.

Reference	Vector	State	Target	Method	Molecular Prevalence ^a^ P/Tested (%)	Typed typ./P (%)	DTU (Number)			
							TcI	TcIV	Other	Mixed Infections
Roellig et al. [32]	*Triatoma sanguisuga*	FL	SL-IR; 24Sα; 18S	PCR; MLEE; RAPD; STR	3/3 (100)	3/3 (100)	3			
	*T. sanguisuga*	GA			1/1 (100)	1/1 (100)	1			
	*Triatoma gerstaeckeri*	TX			3/3 (100)	3/3 (100)	2	1 *		
Cura et al. [45]	*T. gerstaeckeri*	TX	SL-IR	Sequencing	7/7 (100)	7/7 (100)	7			
Hwang et al. [72]	*Triatoma protracta*	CA	24Sα; 18S	Sequencing	34/161 (21)	2/34 (6)			2 TcII/VI	
Herrera et al. [49]	*T. sanguisuga*	LA	SL-IR; 24Sα; 18S	PCR; sequencing	8/12 (67)	6/8 (75)	6			
Buhaya et al. [73]	*Triatoma rubida*	TX	TcSC5D	Sequencing	25/39 (64)	24/25 (96)	24			
Shender et al. [74]	*T. protracta*	CA	SL-IR; 24Sα; HSP60; GPI	PCR; PCR-RFLP	37/97 (38)	22/37 (59)	20	2		
Curtis-Robles et al. [53]	*T. gerstaeckeri*	TX	SL-IR; TcSC5D	MTq-PCR; sequencing	16/16 (100)	16/16 (100)	10	4		2 TcI + TcIV
	*T. sanguisuga*	TX			13/20 (65)	13/13 (100)	2	9		2 TcI + TcIV
Curtis-Robles et al. [54]	*T. gerstaeckeri*	TX	SL-IR	MTq-PCR	1/2 (50)	1/1 (100)	1			
Meyers et al. [55]	*T. gerstaeckeri*	TX	SL-IR	MTq-PCR	9/18 (50)	9/9 (100)	6	1		2 TcI + TcIV
Aleman et al. [52]	*Triatoma lecticularia*	TX	18S	Sequencing	13/19 (68)	13/13 (100)	13			
	*T. sanguisuga*	TX			2/2 (100)	2/2 (100)	2			
Curtis-Robles et al. [75]	*T. gerstaeckeri*	TX	SL-IR; TcSC5D	MTq-PCR; sequencing	574/897 (64)	548/574 (95)	294	189		65 TcI + TcIV
	*Triatoma indictiva*	TX			32/67 (48)	28/32 (88)	9	17		2 TcI + TcIV
	*T. lecticularia*	TX			44/66 (67)	42/44 (95)	9	25		8 TcI + TcIV
	*T. protracta*	TX			3/19 (16)	2/3 (67)	2			
	*T. rubida*	TX			11/64 (18)	7/11 (64)	6	1		
	*T. sanguisuga*	TX			158/315 (50)	135/158 (85)	21	107		7 TcI + TcIV
	*Triatoma* sp.	TX			11/29 (38)	11/11 (100)	4	7		
	*T. sanguisuga*	AL			1/4 (25)	1/1 (100)		1		
	*T. protracta*	AZ			1/7 (14)	1/1 (100)	1			
	*T. rubida*	AZ			5/33 (15)	4/5 (80)	4			
	*T. sanguisuga*	FL			4/25 (16)	4/4 (100)	1	3		
	*T. sanguisuga*	IN			1/2 (50)	1/1 (100)		1		
	*T. sanguisuga*	KS			1/1 (100)	1/1 (100)		1		
	*T. sanguisuga*	LA			3/3 (100)	3/3 (100)	1	2		
	*T. sanguisuga*	MO			1/2 (50)	0/1 (0)				
	*T. rubida*	NM			2/7 (29)	1/2 (100)	1			
	*T. sanguisuga*	OK			1/2 (50)	1/1 (100)		1		
	*T. sanguisuga*	TN			1/2 (50)	1/1 (100)		1		
	*T. sanguisuga*	VA			3/7 (43)	3/3 (100)		3		
Curtis-Robles et al. [76]	*T. gerstaeckeri*	TX	SL-IR	MTq-PCR	1/11 (9)	n.s.	n.s. **			
	*T. protracta*	TX			4/9 (44)	n.s.	n.s. **			
	*T. rubida*	TX			69/299 (23)	n.s.	n.s. **			
Hodo et al. [56]	*T. sanguisuga*	TX	SL-IR; 24Sα; 18S; COII	MTq-PCR	1/4 (25)	1/1 (100)	1			
Dumonteil et al. [77]	*T. sanguisuga*	LA	SL-IR	MTq-PCR; sequencing	40/45 (89)	40/40 (100)	19	3		15 TcI + TcIV 2 TcI + TcII/V 1 TcI + IV + II/V
Rodríguez et al. [67]	*T. rubida*	TX	SL-IR; 24Sα;	PCR	29/50 (58)	27/29 (93)	26	1		
	*T. protracta*	TX			1/2 (50)	1/1 (100)	1			
	*T. gerstaeckeri*	TX			2/2 (100)	2/2 (100)	2			
	*T. rubida*	NM			118/171 (69)	117/118 (99)	109	1		7 TcI + TcIV
Flores-López et al. [78]	*T. gerstaeckeri*	TX	COII-ND1; MSH2; DHFR-TS; TcCLB	MLST	44/n.s.	n.s. ***	75% ***	25% ***		
	*T. lecticularia*	TX			4/n.s.					
	*T. indictiva*	TX			1/n.s.					
	*T. sanguisuga*	TX			2/n.s.					
	*Triatoma recurva*	AZ			4/n.s.					

FL, Florida; GA, Georgia; TX, Texas; CA, California; LA, Louisiana; AL, Alabama; AZ, Arizona; IN, Indiana; KS, Kansas; MO, Missouri; NM, New Mexico; OK, Oklahoma; TN, Tennessee; VA, Virginia; MLEE, multilocus enzyme electrophoresis; RAPD, random amplified polymorphic DNA; STR, short tandem repeats (microsatellites); typ., typed; SL-IR, spliced leader intergenic region of the mini-exon; 18S, 18S ribosomal DNA; 24Sα, 24Sα ribosomal DNA; COII, cytochrome oxidase II; HSP60, heat-shock protein 60; GPI, glucose phosphate isomerase; COII-ND1, cytochrome oxidase subunit II- NADH dehydrogenase subunit 1 region; MSH2, mismatch-repair class 2; DHFR-TS, dihydrofolate reductase-thymidylate synthase; TcCLB, nuclear gene with ID TcCLB.506529.310; TcSC5D, TcSC5D gene; RFLP, restriction fragment length polymorphism; MTq-PCR, Multiplex TaqMan Real-Time PCR; MLST, multilocus sequence typing; n.s., not specified. ^a^ Molecular prevalence was calculated as the number of samples with evidence of *T. cruzi* DNA by means of PCR or culture or direct microscopic examination (positive, P) with respect to the total number of samples analyzed (tested). Molecular prevalences in Roellig et al. [32] do not match reality since positive samples were pre-selected for the study. * TcIV was typified as TcIIa according to the previous nomenclature (see Table 1). ** TcI was the only DTU detected but numbers are not detailed. *** TcI was found in the 75% of the infected triatomines (n = 55). The other 25% showed TcIV-USA, a divergent American branch of TcIV.
